# Temporal RNA-seq analysis reveals previously uncharacterized B cell infiltration during late chronic cerebral toxoplasmosis

**DOI:** 10.3389/fimmu.2026.1885764

**Published:** 2026-10-12

**Authors:** Gabriel Targino, Iasmin Aparecida Cunha Araújo, Anna Laura de Jesus Gomes, Duarte Eduardo Pereira, Valdemir Moreira, Paulo Czarnewski, Neide Maria Silva

**Affiliations:** 1 Laboratory of Immunopathology, Institute of Biomedical Sciences, Federal University of Uberlândia, Uberlândia, Minas Gerais, Brazil; 2 Precisium AI, Solna, Sweden

**Keywords:** B cell, brain, chronic toxoplasmosis, encephalitis, RNAseq, *Toxoplasma gondii*

## Abstract

Chronic toxoplasmosis begins when tachyzoites cross the blood–brain barrier and enter the Central Nervous System (CNS). There, they differentiate into bradyzoites, which persist in tissue cysts. Here, we investigate changes in host gene expression in the mouse brain by RNA sequencing at different time points during chronic toxoplasmosis. We show a conserved gene expression pattern caused by the infection, primarily associated with cytotoxic immune responses against the parasite and neuronal degeneration, regardless of the time point. Surprisingly, we observed that genes associated with B cell function continuously increased over time, whilst cytotoxic responses decreased. Immunoglobulin gene expression and IgA staining showed that B cells were absent from healthy brains. Following infection, they accumulated near penetrating vessels and infiltrated the brain parenchyma. Additionally, these plasma cells produced *T. gondii*-specific IgA. Our results show a previously unrecognized expansion of brain B cells in late chronic toxoplasmosis.

## Introduction

*Toxoplasma gondii* is a neurotropic pathogen with worldwide distribution. In humans, the estimated global seroprevalence of *Toxoplasma gondii* infection is 25.7% (1). *In vitro*, *T. gondii* can grow in human astrocytes, neurons (2), and microglia (3). Studies of primary mouse brain cultures demonstrated that *T. gondii* can infect neurons, astrocytes, microglia, and oligodendrocytes (4, 5). Mouse *in vivo* studies showed that *T. gondii* primarily infects neurons (6). The interferon-γ-dependent immune responses control the rapid parasite growth in the acute phase of infection. Thus, the parasite disseminates to different tissues and differentiates into semidormant cysts mainly within muscle cells and neurons, where they persist for life in the infected host (7). Before effective antiretroviral therapy, *T. gondii* was the most common cause of encephalitis in AIDS patients due to the recrudescence of latent chronic infection (8). However, it remains a health problem if prophylaxis is not provided to seropositive individuals who experience antiretroviral therapy failure (9). Toxoplasmic encephalitis can also develop in other immunosuppressive conditions, such as those following organ transplantation or occurring in patients with cancer receiving immunosuppressive therapy (10).

*T. gondii* triggers innate immunity by inducing IL-12 production by macrophages, dendritic cells, and neutrophils. This promotes NK, CD4, and CD8 T cell activation and interferon gamma (IFN-γ) production, which restrict parasite growth (11). Adaptive immunity to *Toxoplasma* depends on both CD4 and CD8 T cells, with the latter playing a dominant role in controlling chronic infection. Although CD8 T cells play an important effector role in controlling chronic infection, their maintenance depends on help from CD4 T cells (12). Related to B cells, their protective role has not been extensively studied; however, studies utilizing B cell-deficient mice have shown that antibodies may function protectively *in vivo* by blocking tachyzoite infection of host cells (13).

Several meta-analyses have linked T. gondii infection with cognitive and neuropsychiatric disorders, including bipolar disorder, schizophrenia (14), obsessive-compulsive disorder, epilepsy (15), and Alzheimer’s disease (16). However, these associations remain controversial. In mice, chronic *T. gondii* infection also induces behavioral alterations (17). Nevertheless, findings from both human and mouse studies remain controversial, with some studies showing correlations between neuropsychiatric disorders and *T. gondii* infection and others showing no such correlations (18). Thus, the interaction between *T. gondii* and the central nervous system, and the consequences of this interaction, are not completely understood.

Genome-wide transcriptomic profiling provides an unbiased approach to studying normal and pathological conditions. Previous studies have described the transcriptomic changes in the brain during *T. gondii* infection (19–21), primarily focusing on comparisons against healthy controls or contrasting acute versus chronic infection.

In the present study, we performed a detailed longitudinal analysis of differentially expressed genes in the brains of chronically *T. gondii*-infected mice compared with uninfected mice. We describe the temporal changes in gene expression that occur during chronification of infection, highlighting the unexpected detection of immunoglobulin transcripts in late chronic infection. Validation experiments confirmed the recruitment of IgA+ B cells into CNS tissue during chronic toxoplasmosis.

## Results

### Exploration of cerebral toxoplasmosis by RNA sequencing

To characterize the temporal dynamics of gene expression in the brain during chronic infection without bias, we first analyzed a public dataset, GSE117504 (22) (**Figure 1a**). It encompasses a comprehensive collection of transcriptomic data derived from experimental cerebral toxoplasmosis, providing a valuable resource for understanding the intricate molecular mechanisms underpinning this complex disease. It comprises a diverse array of samples representing various time points during infection. Notably, the dataset exhibited robust data quality, as evidenced by over 20 million reads per sample (**Supplementary Figure S1A**) and consistently stable relative log expression (RLE) values after count normalization (**Supplementary Figure S1B**). These results indicate that GSE117504 has high overall sample quality, with no strong batch effects detected.

**Figure 1:**
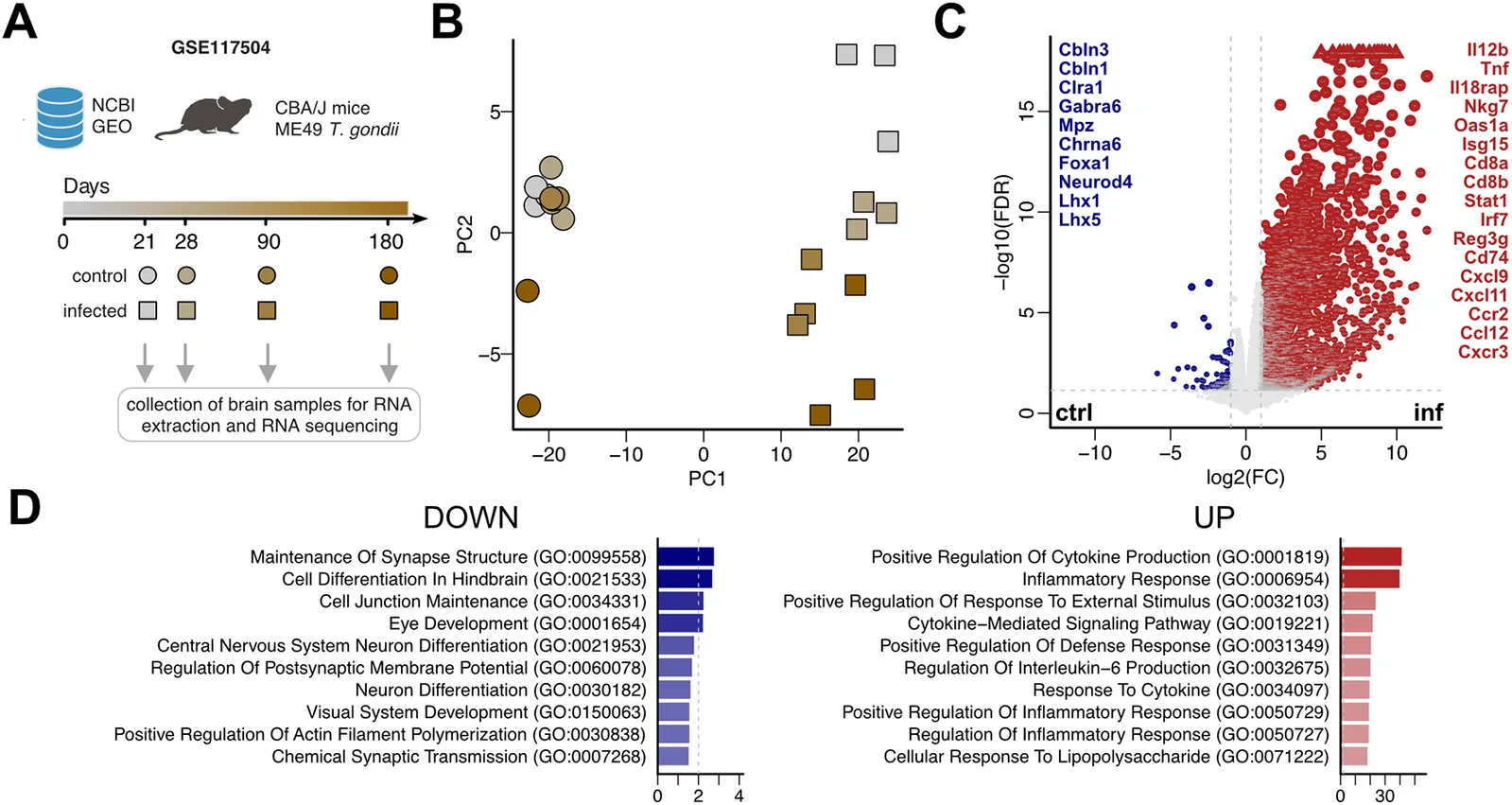
Analysis of bulk RNA sequencing data of chronic cerebral toxoplasmosis. The RNA-seq dataset from GSE117504 (22) was generated using the CBA/J mouse strain (three mice per group). **(A)** Schematic illustration of the experimental design used for collecting brain samples from infected and uninfected mice under chronic toxoplasmosis. Time points are indicated with two to three mice per group. **(B)** Principal component analysis of all samples, highlighting the separation of infected samples (squares) from controls (circles) and the infection duration (color gradient). **(C)** Volcano plot for the conserved signature under infection across all time points, showing significantly up- (red) and downregulated genes (blue). Genes with a −log10(*p*-value) above 2 and log2(FC) above ± 1 (dashed lines) are considered significant. A few selected genes are highlighted. **(D)** Functional gene set analysis for up- and downregulated genes using Gene Ontology. Together, these analyses show that chronic *T. gondii* infection induces a robust and persistent immune transcriptional program while simultaneously suppressing genes associated with neuronal function. Terms with a −log10(*p*-value) above 2 (dashed lines) are considered significant.

Next, we computed gene-wise variance on the normalized counts to identify genes, without bias, whose expression may vary over time and with infection status (**Supplementary Figure S1C**). The top 500 variable genes were selected for unsupervised dimensionality reduction via principal component analysis (PCA) for visualization and data exploration (**Figure 1B**). This analysis revealed a distinct segregation of samples primarily driven by infection status along the first principal component (PC1), which accounted for over 80% of all variation in the dataset. In contrast, the duration of infection was the main driver of separation along the second principal component (PC2), with about 5% of the variance explained (**Figure 1B**). This compelling separation strongly implies the pivotal role played by infection in shaping the intricate molecular landscape associated with cerebral toxoplasmosis and suggests that the chronic infection is not homogeneous.

To further dissect the effect of infection and distinguish it from the effect of infection duration, we employed a generalized linear model (GLM) with edgeR (23), a flexible statistical method that allows the effects of infection and time to be separated. In other words, GLMs consider the gene expression of any given gene to be a combination of the effects of the variables (in this case, infection and time). We modeled the gene expression as the complete interaction between the infection status and the time points, including all interactions of the infection at different days of infection:


EXP ∼ infection + time + infection:time

With this approach, we identified more than 2,500 differentially expressed genes (DEGs) in infected brains versus uninfected controls, regardless of the time of infection (**Figure 1C**). This large number of infection-induced DEGs is consistent with the PCA results, in which infection contributed the most to PC1. The upregulated gene cluster was notably associated with pathways involved in cytokine signaling (*Il12b*, *Tnf*, *Il18rap*), type 1 immune response (*Nkg7*, *Oas1a*, *Isg15*, *Cd8a/b*, *Stat1*, *Irf7*), response to microbial stimuli (*Reg3g*, *Cd74*, *Tnf*), and cell chemotaxis (*Cxcl9*, *Cxcl11*, *Ccr2*, *Ccl12*, *Cxcr3*), providing insight into the complex host immune response to cerebral toxoplasmosis (**Figure 1D**). Conversely, the downregulated genes were associated with pathways governing synaptic structure (*Cbln3*, *Cbln1*), chemical synaptic transmission (*Glra1*, *Gabra6*, *Mpz*, *Chrna6*, *Cbln1*), and neuronal development (*Foxa1*, *Neurod4*, *Lhx1*, *Lhx5*), suggesting potential disruptions in neuronal signaling pathways induced by the infection (**Figure 1D**). The complete list of differentially expressed genes and pathway enrichment analyses is provided in**Supplementary Tables S1**, **S2**. These findings provide insight into the complex interplay between the host immune response and neurophysiological alterations in the context of cerebral toxoplasmosis.

### B cell-related genes are enriched in late chronic cerebral toxoplasmosis

Having identified the top DEGs driven by *T. gondii* infection using the GLM, we next investigated the heterogeneity of chronic infection, as reflected in PC2 previously described (**Figure 1B**). We therefore computed differential expression across time points, including samples from early chronic infection (days 21 and 28) and late chronic infection (days 90 and 180). Compared with day 21, when the first tachyzoites arrive and start colonizing the brain (24), samples from day 28 showed only a handful of genes differentially expressed (11 upregulated and three downregulated) (**Figure 2A**). Surprisingly, samples from days 90 and 180 of infection showed much larger numbers of DEGs, including both up- and downregulated genes (**Figure 2A**). We also noted that many of the genes identified across the time points were significantly dysregulated, although to different extents. These include genes related to reduced mitotic spindle activity (*Cdc20*, *Cenpf*, *Nuf2*, *Bub1b*, *Ttk*) and diminished T cell immunity (*Trbc1*, *Nkg7*, *Cd3g*, *Cd3e*, *Cd3d*) and NK cytotoxicity (*Il21*, *Ncr1*, *Klrk1*, *Klrc2*, *Klrd1*, *Klrc1*) (**Supplementary Figure S2**). These results highlight that, after the early chronification phase (around days 21 to 28), the abundance of T cells and other immune cells decreased. This pattern is in line with the interconversion of tachyzoites to bradyzoites and parasite evasion and immune response (25) and with the regulation of early mitotic activity during bradyzoite differentiation (26).

**Figure 2:**
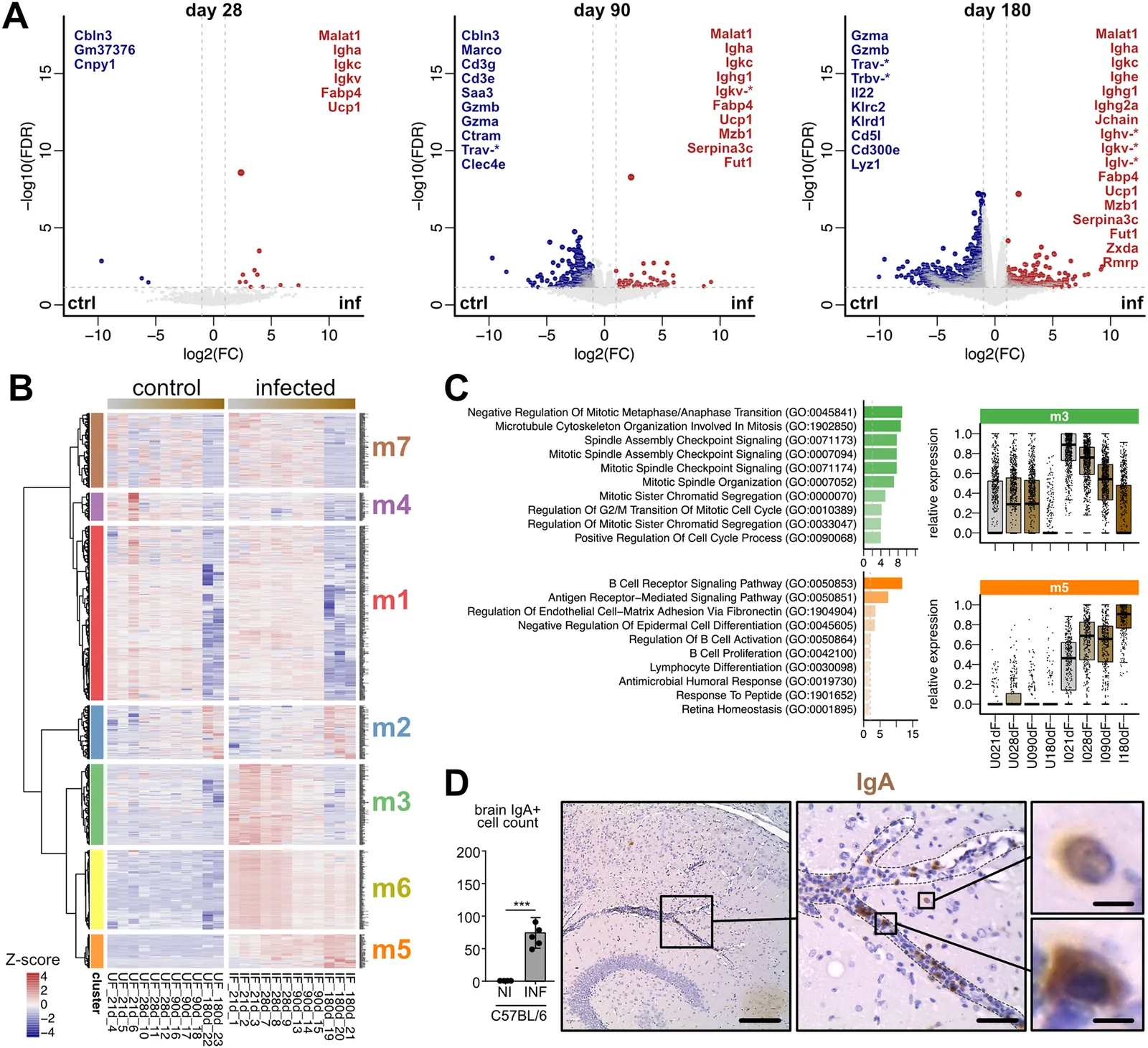
B cell dynamics in the brain during chronic *T. gondii* infection. The RNA-seq dataset from GSE117504 (22) was generated using the CBA/J mouse strain (three mice per group). **(A)** Volcano plots for genes that are differentially expressed at each time point, compared with mice at day 21 of infection. Selected up- and downregulated genes are shown. **(B)** Heatmap for all genes temporally affected by the infection, which were clustered into seven distinct gene modules (m1–m7). The color represents the scaled gene expression across samples (*Z*-score). **(C)** Temporal expression trend and gene set analysis for selected gene modules m3 and m5. Each dot represents the normalized expression of a gene within the gene module for each sample group. **(D)** Immunohistochemistry staining for IgA in the mouse brain at 30 days of infection (BALB/c strain). These results identify a progressive enrichment of B cell-associated genes during late chronic infection and experimentally confirm the presence of IgA+ B cells within the infected brain parenchyma. The region delimited by the dashed gray line represents the choroid plexus. Scale bars represent 200, 40, and 5 µm. Boxplots represent the fifth, 25th, 50th, 75th, and 95th quantiles, respectively.

Among genes upregulated on days 90 and 180 of infection, genes associated with B cell function were identified, including B-cell differentiation (*Mzb1*, *Cd79a/b*), class-switched antibody chains (*Igha*, *Ighe*, *Ighga*, *Ighg2c*, *Jchain*), and several B cell receptor genes (*Igkv*, *Ighv*, *Igkc*, *Ighc*) (**Figures 2A, B**). These findings are particularly surprising because, to the best of our knowledge, B cell expansion or migration to the brain during *T. gondii* infection appears to be a previously unrecognized phenomenon. Similar phenomena have been reported in other pathologies, particularly when *Cxrc3* and *Cxcl9* are produced in the brain, promoting B cell diapedesis (27). In fact, both of these molecules were among the top DEGs found in chronically infected brains (**Figure 1C**).

### B cells migrate to the brain in late chronic infection

We next decided to investigate further whether the findings above could be driven purely by natural aging of the brain or by the maturation of the immune response during *T. gondii* infection. To this end, we selected all genes differentially expressed at any given time point and clustered them into temporally coherent gene modules (**Figures 2B, C**; **Supplementary Figure S3**). Among these modules, three stood out: a module enriched for genes involved in the T cell immune response (m6), a module enriched for B cell signatures and activation (m5), and a module enriched for the control of cell division (m3) (**Figure 2C**). Importantly, only module m2 showed an expression pattern enriched in 180-day-old mice (regardless of infection status), suggesting a gene network associated with senescence/maturation. Conversely, modules m1 and m7 showed decreased or nearly absent expression only at day 180, regardless of infection status. However, none of these modules (m1, m2, and m7) showed significant pathway enrichment (**Supplementary Figure S3**). Module m4 also showed a gene expression trend that decreased over time regardless of infection status, with genes associated with cranial skeletal development and neuronal maturation. These findings are in line with previous studies showing that molecular development in the brain (28) and structural (29) development peak at around 30–60 days of age and decline thereafter.

The B cell module m5 had no genes detected in uninfected mice, suggesting that B cell expansion is a phenomenon triggered by inflammation (**Figure 2C**). Thus, we sought to directly verify whether IgA+ B cells are indeed present in the tissue as early as day 30 of infection, a time point commonly used for the study of chronic toxoplasmosis. In fact, staining for IgA+ cells in the brain of infected mice at 30 d.p.i. showed that B cells were found primarily near the vessels of the pia mater (**Figure 2D**) but could also infiltrate the parenchyma (**Figure 2D**). Quantification of IgA+ cells in the healthy brain showed little to no detection of B cells (**Figure 2D**), further corroborating the RNA-seq results regarding the near absence of B cells in the healthy brain. We observed that IgA+ cells were located throughout the sections and were not grouped into inflammatory foci or tertiary lymphoid structures, consistent with our hypothesis that IgA+ cells migrate to the tissue rather than arise from local expansion of tissue-resident B cells. It is plausible that B cells at day 180 might be transcriptionally distinct from those found at day 30, but this distinction could be further characterized in future studies using single-cell sequencing. In summary, we showed through quantification of B cell transcripts in an RNA-seq dataset and direct detection of IgA+ cells in the brain that B cells are nearly absent from the healthy brain and can infiltrate the cerebral parenchyma during infection.

### These IgA-positive cells are experts in producing specific-*T. gondii* antibodies

To get an idea of whether this IgA is protective against infection, we evaluated the specificity and levels of IgA in the brain and systemically in two mouse strains, susceptible C57BL/6 and resistant BALB/c, to *T. gondii* infection. Female and male C57BL/6 mice showed severe inflammation in the brain, which was characterized by vascular cuffing, inflammatory foci in the parenchyma, and inflammatory cell infiltration in the meninges, while the lesions in the brain of BALB/c mice were milder (**Figure 3A**). Both mouse strains showed IgA+ cells in the parenchyma (**Figure 3B**), and these cells produced *T. gondii*-specific IgA, which was higher in susceptible mice than in resistant mice, although the difference did not reach statistical significance (**Figure 3C**). Interestingly, both mouse strains produced specific IgA systematically, whose levels showed an opposite pattern to those observed in the brain (**Figure 3D**). These data suggest that brain-specific IgA does not appear to protect the brain of C57BL/6 mice against infection and inflammation.

**Figure 3:**
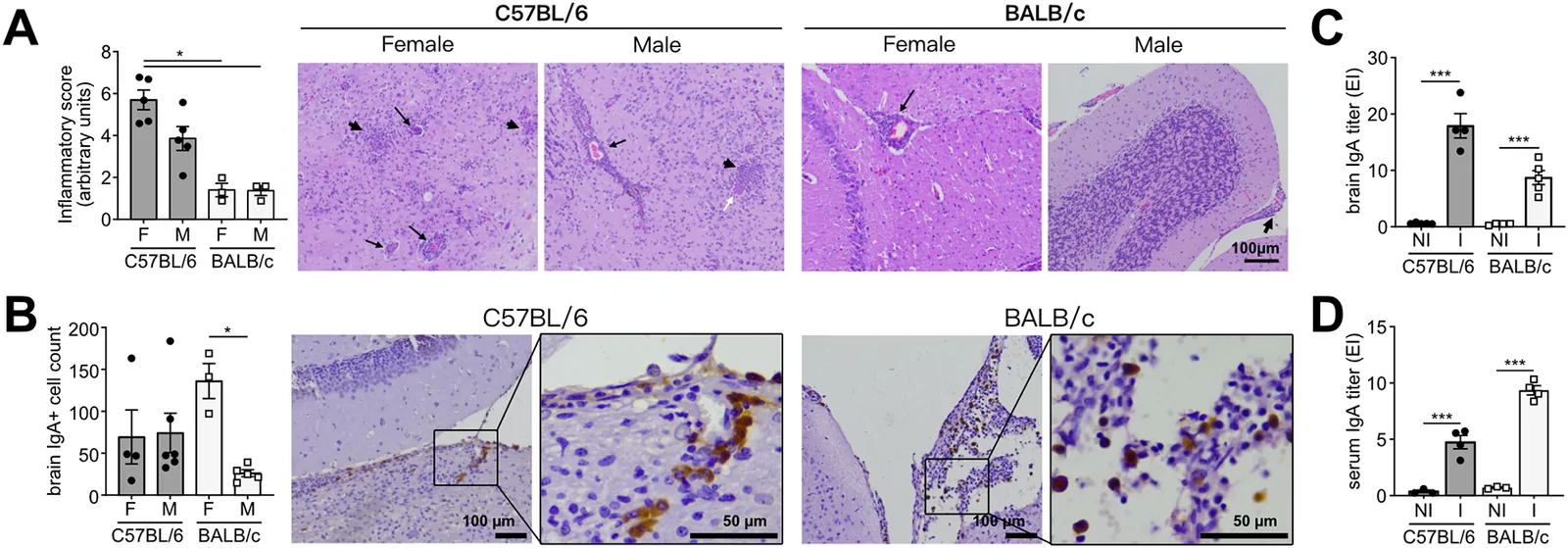
Both infected C57BL/6 and BALB/c mouse strains showed IgA+ cells in the brain and specific IgA in serum samples following infection. Animals were infected by the intraperitoneal route, and on day 30 of infection, the brain and serum samples were collected, and specific IgA was analyzed by ELISA. **(A)** Inflammatory score and photomicrographs of infected brains (H&E staining). Inflammatory foci (arrowheads), vascular cuffing (black arrows), inflammatory cells in the meninges (large arrow), and a cyst-like structure (white arrow) were observed in the parenchyma of infected animals. **(B)** IgA+ cells detected by immunostaining. **(C)** *T. gondii*-specific IgA antibodies in the brain homogenate and **(D)** serum samples detected by ELISA. Although both mouse strains exhibited IgA+ cells in the brain, susceptible C57BL/6 mice developed more severe neuropathology, indicating that the presence of IgA+ cells alone is insufficient to prevent brain inflammation. Data are representative of one of two independent experiments with five mice per group and are presented as mean ± SEM. Differences were analyzed by one-way ANOVA followed by Šídák’s multiple comparisons test. ^*^p < 0.05; ^**^p < 0.01; ^***^p < 0.001.

## Discussion

*T. gondii* infection induces an innate immune response that represents the first line of defense against the invading pathogen; however, the parasite has evolved strategies to evade this response and persist as a chronic infection mainly in the CNS, with the adaptive immune response being essential for the ultimate survival of the infected host (30). To identify correlates of immunity, we analyzed the genome-wide brain transcriptome profiling dataset obtained from the Garfoot et al. study (22) to identify genes differentially expressed in the brains of chronically *T. gondii*-infected mice on days 28, 90, and 180 of infection. An important limitation of this study is that the transcriptomic analyses are based on a reanalysis of a publicly available RNA-seq dataset. Consequently, we were limited to the experimental design, sample collection, and metadata generated in the original study and could not perform additional transcriptomic analyses or control for potential unmeasured confounding factors. Moreover, bulk RNA-seq cannot determine the spatial distribution or functional role of the identified cell populations. To address this limitation, we experimentally validated our principal finding of IgA+ B cell infiltration into the infected brain, although the remaining transcriptomic observations warrant further independent validation.

Samples from days 90 and 180 of infection presented much higher numbers of DEGs, which were related to the mitotic spindle of the host cells, T cell immunity, and NK cytotoxicity. As an intracellular parasite, *T. gondii* manipulates a broad range of host cellular functions, such as host cell division. The published data related to the cell cycle are controversial. Some studies using immortalized human foreskin fibroblasts indicate an infection-driven shift from G0/G1 to S phase, with accumulation of host cells in S phase (31, 32), whereas a study using a human trophoblast cell line (BeWo) reported arrest in the G2/M phase (33), thereby most likely reflecting cell type-specific reactions. In primary bovine umbilical vein endothelial cells (BUVEC), *T. gondii* dysregulates the host cell cycle, causing G2/M arrest through functional alterations, such as chromosome segregation errors, mitotic spindle alteration, and blockage of cytokinesis (34). Thus, consistent with previous *in vitro* studies, these findings from the transcriptomic analysis suggest that *T. gondii* is able to alter the cell cycle in the infected brain.

The role and activation of T lymphocytes in chronic *T. gondii* infection are well characterized (35, 36). Related to NK cells, it has been shown that *NKG2D* on NK cells plays a critical role in the NK-DC interaction, which is important for the generation of robust CD8+ T cell immunity to *T. gondii* infection, protecting mice against the high parasite load in the brain (37). However, NK cells negatively regulate CD8 T cells, promoting their immune exhaustion and the persistence of the parasite and chronic infection in the brain (38). Thus, the long-term interactions between the parasite and the host in late chronic infection are associated with decreased transcripts of T lymphocytes and NK cells in the organ that is most affected in late chronic infection.

Related to B cells, their role in *T. gondii* infection is less understood, particularly in the CNS. Previous studies have shown that B cell-deficient mice (muMT) infected with the ME-49 strain by the oral route survived acute infection; however, they died in early chronic infection, with parasite replication and pathology in their brains (39). Additionally, even after vaccination with the ts-4 RH strain, muMT mice were highly susceptible to challenge with the RH strain, in contrast to wild-type mice (13). In both experiments, the susceptibility of B cell-deficient mice did not appear to be caused by deficient T cell functions or diminished capacity to produce IFN-γ. Interestingly, when muMT mice were orally infected with the Fukaya strain and received an intraperitoneal transfer of B1 cells, but not B2 cells, from primed *T. gondii*-infected B6 mice, they survived for more than 5 months (40). Nevertheless, it has been shown that the humoral immunity to *T. gondii*, regulated by Nfkbid, a nuclear regulator of NF-κB, is required for full protection against the parasite and requires B-1 and B-2 cells (41).

Thus, B cells are involved in protection against *T. gondii* infection mainly in the chronically infected brain. The Garfoot et al. (22) dataset of brain transcriptome analyzed herein showed that genes associated with B cell differentiation were upregulated on days 90 and 180, whereas genes encoding class-switched antibody chains were upregulated from day 28 onward. Among the genes encoding class-switched antibody chains expressed in the brain of chronically infected mice were *Igha*, *Ighe*, *Ighga*, *Ighg2c*, and *Jchain*.

There is evidence that antibodies may function protectively *in vivo* against *T. gondii* infection. *Toxoplasma*-immune serum improved the resistance of ts-4-vaccinated-muMT (13) or CD4-deficient (42) mice after a challenge with virulent RH tachyzoites. In both cases, the immune serum contained *T. gondii*-specific *IgG* isotypes. B cell-deficient (immunoglobulin M-suppressed from the neonatal period) mice were highly susceptible to *T. gondii* challenge; however, the transfer of anti-*Toxoplasma* Ab induced partial protection, mainly in chronic infection (43).

Related to IgA, this is an antibody isotype predominantly produced at mucosal surfaces; thus, the *IgA* class-switched antibody chain gene expression in the CNS in chronic *T. gondii* infection was somewhat surprising. Thus, we infected mice with *T. gondii* and examined the brain on day 30 of infection to search for IgA+ cells in the organ. IgA+ cells were observed in the brains of both highly susceptible C57BL/6 mice and resistant BALB/c mice to infection. Additionally, the IgA produced in the brain is *T. gondii*-specific. It was shown that both mouse strains had IgA+ cells that were located in the vicinity of meningeal vessels. The observations described above should therefore be considered possible biological explanations rather than conclusions supported by the present study. Our experiments were not designed to determine the origin or functional activity of these IgA+ cells.

The role of *T. gondii*-specific IgA production locally is still unclear and remains to be demonstrated in future studies. We speculate that its function could be analogous to those described in other pathogens and diseases. The majority of B cells in the dura are B2 cells, and a large fraction of these are immature B cells generated in the skull’s bone marrow (44). Some B cells in the dura are IgA+ cells (45). Under steady-state conditions of meningeal humoral immunity, it was shown that in the unchallenged meninges, specifically in the mouse dura mater, most of the peri-sinus plasma cells were IgA-positive (46). Additionally, IgA+ plasma cells were also almost completely absent in the meninges of germ-free (GF) mice, but their presence was restored in GF mice reconstituted with either a murine or human gut microbiome, or infected with the enteropathogenic *Citrobacter rodentium*, suggesting that these cells might arise from B cells in the gut and differentiate into plasma cells in the dura, or that plasma cells might differentiate in the gut and migrate to the dura (46). In IgA-deficient mice infected with *Candida*, increased fungal spread into the brain parenchyma was observed, confirming the importance of IgA+ cells in the meninges (46). Thus, the IgA+ cells might migrate from the gut to the dura and be protective against pathogens in the CNS. Consistent with this possibility, Rojas et al. showed the presence of gut-derived IL-10-producing IgA+ plasma cells within the brain and spinal cord during inflammation in a mouse model of multiple sclerosis (experimental autoimmune encephalomyelitis [EAE]), and the removal of plasmablasts and/or plasma cells resulted in exacerbated EAE, which was normalized by the introduction of gut-derived IgA+ plasma cells (47). In the present study, we speculate that the IgA+ cells detected in the brain of chronically infected mice could be generated in the skull’s bone marrow and migrate to the meninges or could arise from the periphery. However, these IgA+ cells did not appear to be protective in highly susceptible C57BL/6 mice, as these mice presented substantial inflammatory changes and tissue parasitism in the brain (data not shown). Whether the IgA+ cells identified in our study share this origin or perform similar functions remains unknown.

The serological detection of *T. gondii* IgA has been used as a diagnostic biomarker of ocular (48) and recent infection in pregnant women (49). Several studies have detected IgG isotypes and IgA subclasses systemically and in the feces using different vaccination protocols involving intranasal, intramuscular, or oral routes and influenza virus-like particles (VLPs) or recombinant vaccinia viruses (rVVs) expressing different *T. gondii* antigenic proteins or recombinant *T. gondii* proteins (36, 39, 50–52). Additionally, we and others have detected IgA in the feces of mice orally infected with *T. gondii* (53–55). However, the effective role of IgA in the periphery or CNS during infection remains unclear. It has been shown that SIgA from human milk was able to inhibit replication in enterocytes *in vitro* (56). Our work provides evidence that B cells infiltrate the infected brain and locally produce *T. gondii*-specific IgA. Although this finding expands our current understanding of chronic cerebral toxoplasmosis, the functional significance of these cells remains to be determined.

In the brain parenchyma, only one study showed the detection of *T. gondii-*specific IgA in brain homogenates of mice infected or immunized and infected with *T. gondii* (57); however, the detection of IgA+ cells in the brain of mice infected with *T. gondii* has not been clearly demonstrated, to the best of our knowledge. In fact, current publications on cerebral toxoplasmosis do not mention B cells in the brain (58–60). This includes the key work from Weiss et al., “*Toxoplasma gondii* - the apicomplexan model” (Chapter 24) (59), where they explicitly state that whether B cells traffic to the CNS to produce antibodies locally is unknown. Our work contributes to this perspective by providing evidence that B cells are recruited to the brain during infection and produce *T. gondii*-specific IgA, a phenotype that appears to have been previously unrecognized. Nonetheless, additional experiments are necessary to elucidate the origin of the IgA+ cells and their roles in the chronically infected brain, especially using single-cell or spatial omics to explore potential novel cell interactions, spatial niches, and B cell subsets.

## Conclusion

By analyzing a public dataset on toxoplasma encephalitis, we showed that *T. gondii* is associated with a clear inflammatory response in the brain. Surprisingly, pathways related to the cell cycle, type I immune response, and B cell-related genes showed temporal changes during the chronification of infection. Finally, we validated that B cells are rarely present in the healthy brain but are able to infiltrate the parenchyma upon *T. gondii* infection.

## Materials and methods

### Transcriptomic analysis

Data analysis was conducted using the R programming language within the RStudio environment. The collected datasets were normalized and combined into a single matrix to enable analysis of specific genes. Of the datasets obtained, only the dataset from Garfoot et al. (24) (GSE117504) was included in accordance with our study objective. Differential gene expression was carried out with edgeR (23), using a design matrix that modeled infection, time points, and all corresponding interactions (see Results). Genes with an absolute fold change above two and an adjusted false discovery rate (FDR) below 0.05 were considered significant in all tests. Temporal gene module identification was performed using hierarchical clustering, as previously described (61). Genes detected in each module were then subjected to functional annotation by gene set analysis (GSA) using EnrichR (62), where pathways with *p*-values below 0.01 were considered statistically significant. Data were visualized using bar plots, boxplots, principal component analysis (PCA) scatter plots, heatmaps, and volcano plots.

### Animals

Eight-week-old female C57BL/6 and BALB/c mice were maintained at the Universidade Federal de Uberlândia animal facility (Rede de Biotérios de Roedores [REBIR/UFU]), with 12-h light/dark cycles and free access to food and filtered water. All animal experiments were performed in accordance with the Brazilian Government’s ethical guidelines and were approved by the Comitê de Ética na Utilização de Animais (CEUA) of the Universidade Federal de Uberlândia, under number 011/21.

### Experimental procedure

*T. gondii* ME49 strain was maintained in *Calomys callosus* (Rodentia: Cricetidae), which were inoculated intraperitoneally with 10 cysts of *T. gondii*. One month after infection, ME-49 cysts were harvested from the brains of the animals, quantified, and adjusted to 10 cysts in 0.2 mL of phosphate-buffered saline (PBS; 0.1 mol/L, pH 7.2) to infect experimental mice by the intraperitoneal route (i.p.).

BALB/c and C57BL/6 male and female mice were infected i.p. with 10 cysts of ME49 *T. gondii*. Mouse survival, weight, and morbidity were recorded during the experiment (**Supplementary Figure S4**), as previously described (63). On day 30 after infection, groups of mice were anesthetized with ketamine 100 mg/kg (Syntec Brasil Ltda, Cotia, SP, Brazil) and xylazine 10 mg/kg (Schering-Plough Coopers, Cotia, SP, Brazil) by the intraperitoneal route and were euthanized by cervical dislocation. Blood samples were collected for serological assays, and brains were collected, fixed in 10% buffered formalin, and processed routinely for paraffin embedding and sectioning. Additionally, brain tissue samples were collected, frozen immediately, and stored at − 80 °C for specific IgA detection by enzyme-linked immunosorbent assay (ELISA).

### Histological processing and IgA detection

The brain tissue sections were 4 μm thick and were stained with hematoxylin–eosin (H&E) for histological analysis. Images of the tissues were obtained using an Aperio AT Turbo scanner (Leica Biosystems Imaging, United States) that enabled whole-tissue image management, or the tissues were examined using a light microscope.

The IgA-positive cells were evaluated in the brain by immunohistochemistry. To detect IgA+ cells, deparaffinized tissue sections were placed in a humidified chamber; endogenous peroxidase activity was blocked with 3% H_2_O_2_, and the antigen retrieval was performed in citrate buffer (pH 6.0) in a microwave oven for 7 min. Nonspecific binding was reduced by incubating the slides with 0.3% nonfat milk in PBS (EAN7891000101520, Molico, Nestlé, São Paulo, SP, Brazil) at room temperature. The sections were permeabilized using 0.01% Triton X-100 and incubated at 4 °C overnight with goat antimouse IgA (M8769, Sigmahemic-Aldrich, Saint Louis, MO, USA) diluted in 0.01% saponin. Sections were then incubated with biotinylated donkey antigoat antibodies (705-065-147, Jackson ImmunoResearch Inc., West Grove, PA, USA). The assay sensitivity was improved by the addition of an avidin–biotin complex (PK-4000, VECTASTAIN ABC Kit, Vector Laboratories, Inc., Burlingame, CA, USA) diluted 1:100. The reactions were developed using 3,3′-diaminobenzidine tetrahydrochloride (Sigma). The sections were counterstained with Harris hematoxylin and examined under a light microscope using a × 40 objective to detect tissue parasitism. The IgA+ cells were counted in each brain tissue section. Two histological sections for each mouse, 40 μm apart, were examined and quantified in a double-blind manner by two independent researchers.

### *T. gondii-*specific IgA detection in brain homogenate and serum samples by ELISA

An indirect ELISA was carried out to detect IgA antibodies to *T. gondii* soluble antigen [STAg, prepared as previously described (64)]. The polystyrene microtiter plates (Kartell SPA, Novigilio, Milan, Italy) were coated overnight at 4°C with STAg at 10 μg/mL in 0.06 M carbonate buffer. The mouse serum samples were diluted in Tween-20 PBS (PBS-T) plus 1% nonfat milk (EAN7891000101520, Molico, Nestlé) (PBS-TM), and undiluted brain homogenate was added and incubated for 1 h at 37°C. After washing, the plates were incubated for 1 h at 37°C with peroxidase-labeled antimouse IgA antibodies (sc-3791, Santa Cruz Biotechnology, Santa Cruz, CA, USA). The reaction was developed with o-phenylenediamine (OPD) (P9029, Sigma-Aldrich). The optical density (OD) was measured at 492 nm using a plate reader (Versa Max Microplate Reader, Molecular Devices, Sunnyvale, CA, USA). The mean plus three standard deviations (SD) of OD values for sera from noninfected mice was used to define the ELISA cutoffs for negative serum samples.

## Data Availability

Publicly available datasets were analyzed in this study. This data can be found here: GEO (GSE117504).
